# Neighborhood deprivation and coronary heart disease in patients with bipolar disorder

**DOI:** 10.1038/s41598-022-21295-0

**Published:** 2022-10-06

**Authors:** Filip Jansåker, Veronica Milos Nymberg, Jan Sundquist, Kenta Okuyama, Tsuyoshi Hamano, Kristina Sundquist, Xinjun Li

**Affiliations:** 1 Center for Primary Health Care Research, Lund University, Skåne University Hospital, Jan Waldenströms Gata 35, 205 02 Malmö, Sweden; 2 Department of Clinical Microbiology, Center of Diagnostic Investigations, Copenhagen University Hospital, Rigshospitalet, Denmark; 3 Department of Family Medicine and Community Health, Department of Population Health Science and Policy, Icahn School of Medicine at Mount Sinai, New York, USA; 4 Center for Community-Based Healthcare Research and Education (CoHRE), Organization for Research and Academic Information, Shimane University, Shimane, Japan; 5 Department of Sports Sociology and Health Sciences, Kyoto Sangyo University, Kyoto, Japan

**Keywords:** Cardiology, Risk factors, Disease prevention, Health policy, Public health, Bipolar disorder

## Abstract

The aim was to study the potential effect of neighborhood deprivation on incident and fatal coronary heart disease (CHD) in patients with bipolar disorder. This was a nationwide cohort study which included all adults aged 30 years or older with bipolar disorder (n = 61,114) in Sweden (1997–2017). The association between neighborhood deprivation and the outcomes was explored using Cox regression analysis, with hazard ratios (HRs) and 95% confidence intervals (CIs). Patients with bipolar disorder living in neighborhoods with high or moderate levels of deprivation were compared with those living in neighborhoods with low deprivation scores. There was an association between level of neighborhood deprivation and incident and fatal CHD among patients with bipolar disorder. The HRs were 1.24 (95% CI 1.07–1.44) for men and 1.31 (1.13–1.51) for women for incident CHD among patients with bipolar disorder living in high deprivation neighborhoods compared to those from low deprivation neighborhoods, after adjustments for potential confounders. The corresponding HR for fatal CHD were 1.35 (1.22–1.49) in men and 1.30 (1.19–1.41) in women living in high deprivation neighborhoods. Increased incident and fatal CHD among patients with bipolar disorder living in deprived neighborhoods raises important clinical and public health concerns.

## Introduction

Bipolar disorder is a severe chronic mood disorder^1^ associated with several medical conditions contributing to substantial morbidity and mortality^2,3^. Several studies have demonstrated common medical illnesses comorbid with bipolar disorder, including obesity, hyperlipidemia, hypertension, and diabetes mellitus; all of which are recognized as risk factors for morbidity and fatal coronary heart disease (CHD)^4–8^, relative to the general population. There is also strong evidence showing that the socioeconomic status at the individual and neighborhood level shapes people’s cardiovascular risk factors^9–11^. In Sweden, neighborhood deprivation has been strongly associated with CHD in both men and women^12^. Furthermore, it is known that neighborhood-level socioeconomic status is associated with bipolar disorder^13,14^. However, the association between neighborhood deprivation and CHD in patients with bipolar disorder remains to be established. Such knowledge could help identify patients with bipolar disorder at an increased risk of CHD. Therefore, we aimed to assess the association between neighborhood deprivation and incident and fatal CHD in patients diagnosed with bipolar disorder in a nationwide follow-up study.

The aim of the present study was thus to investigate whether there is a difference in the risk of incident and fatal CHD between patients with bipolar disorder living in deprived neighborhoods and patients with bipolar disorder living in affluent neighborhoods, and whether this possible difference remains after accounting for individual-level sociodemographic characteristics and comorbidities. Considering the background evidence^9–14^, the study hypothesis was that neighborhood deprivation would be associated with coronary heart disease in patients with bipolar disorder.

## Methods

### Design, study population, and settings

This was a nationwide cohort study, which utilized data from national, comprehensive registers and nationwide primary healthcare data, containing individual-level information on all people residing in Sweden. The study population consisted of individuals aged 30 years and older with a diagnosis of bipolar disorder. The control group consisted of patients with bipolar disorder living in affluent neighborhoods; they were compared with patients with bipolar disorder living in deprived neighborhoods. The index day (baseline) was when a participant with a bipolar diagnosis was 30 years and older. The study was conducted at the Center for Primary Healthcare Research, Lund University, Sweden.

### Ascertainment study population

We identified 64 035 unique patients with bipolar disorder, according to the 10th edition of the International Classifications of Diseases (ICD-10) codes F30 and F31, during the study period (1997–2017). Of these, we excluded 791 (1.2%) individuals who had previously been diagnosed with CHD priorly (1987–1996) to the study period (ICD-9 codes 410–414) and 2130 (3.3%) individuals who were diagnosed with CHD (ICD-10 codes I20-I25) before the first diagnosis of bipolar disorder during the study period. A total of 61 114 patients with bipolar disorder (95.4% of the original cohort of patients with bipolar disorder) remained suitable for inclusion in the study population.

### Data source

This study used several comprehensive nationwide register and primary healthcare data, which contain individual-level information on all people in Sweden, including on age, sex, socioeconomic status, geographical region of residence, healthcare diagnoses, and dates of hospital admissions, date of emigration, as well as date and cause of death. The medical conditions were collected from nationwide primary healthcare data (1997–2017), from 20 of 21 Swedish administrative healthcare regions, as well as the National Patient Register with Outpatient (specialist care) data (2001–2017) and Inpatient (Hospital) data (1964–2017), managed by the National Board of Health and Welfare (Swedish: *Socialstyrelsen*). The Cause of Death Registers (*Socialstyrelsen*, 1961–2018) and the Total Population Register (*Statistics Sweden, SCB*, 1968–2018), which were nearly complete for the entire national population, were also used.

The linkages were performed using the national unique civic registration number assigned to each person in Sweden upon birth or immigration to the country. This number was replaced by serial numbers to ensure the integrity of all individuals. This approach was also similar to our previous studies on incident and fatal CHD^15–17^.

### Outcome variables

The main diagnoses for CHD recorded in the National Patient Register and Cause of Death Register were collected. In the present study, the first-time hospital admission for CHD was defined as an incident event according to ICD-10 codes I20-I25 during the study period; fatal CHD was collected in the Cause of Death Register.

### Neighborhood-level variable

Neighborhood deprivation was the main exposure and assessed at baseline. The assessment of this variable was possible as the home addresses of all adults living in Sweden have been geocoded to small geographic administrative units that have boundaries defined by homogeneous types of buildings. These neighborhood units, so called small area market statistics (SAMS) have an average of 1000 to 2000 people and were used as proxies for neighborhoods^12^. *Neighborhood Deprivation Index were calculated as* a summary measure and used to characterize neighborhood-level deprivation. We identified deprivation indicators used by past studies to characterize neighborhood environments and then used a principal components analysis to select deprivation indicators in the Swedish national database^18^. The following four variables were selected for those aged 25–64: low educational status (< 10 years of formal education); low income (income from all sources, including that from interest and dividends, defined as less than 50% of individual median income); unemployment (not employed, excluding full-time students, those completing compulsory military service, and early retirees); and social welfare assistance. The calculation of the neighborhood deprivation index was based on the population aged 25 to 64 years since this age group (i.e., the working population) was considered to be more socioeconomically active than other age groups. The study population, however, consisted of individuals aged 30 years and older. All four deprivation variables were loaded on the first principal component with similar loadings (+ 0.47 to + 0.53) and explained 52% of the variation between these variables^19,20^. A Z-score was calculated for each SAMS neighborhood. The Z-scores were weighted by the coefficients for the eigenvectors and thereafter summed to create the index^21^. The index was categorized into three groups: below one standard deviation (SD) from the mean (low deprivation), above one SD from the mean (high deprivation), and within one SD of the mean (moderate deprivation). Higher scores reflect more deprived neighborhoods^19,20^.

### Individual-level variables

All individual-level variables were assessed at the time (year) of the bipolar disorder diagnosis. Separate analyses were conducted for sex (men and women). The sociodemographic variables were assessed at baseline. *Age* was defined as a continuous variable (≥ 30 years). *Educational attainment* was stratified into three groups as follows: < 9 years, 10–11 years or ≥ 12 years of school attendance. *Family income* was based on the annual family income divided by the number of people in the family. This variable was provided by the Swedish Government-owned statistics bureau (Statistics Sweden). The income variable considered the age of each family member and used a weighted system, i.e. the sum of the total family income of all members was multiplied by the individual’s consumption weight (adults were given higher weights than adolescents and children) divided by the family members’ total consumption weight. Country of origin (*Immigration status)* was divided into two groups: born in Sweden and originating from outside of Sweden (i.e., not born in Sweden). *Marital status* was divided into two groups: never married/widowed/divorce and married/cohabitating. *Region of residence* was categorized into large cities (Stockholm, Göteborg, and Malmö), middle-sized towns, and small towns/rural areas. *Comorbidities* common in patients with bipolar disorder and associated with cardiovascular health and neighborhood deprivation were included as adjustment variables^4–8,22–24^*.* The following medical conditions were identified from the National Patient Register during the study period as follows: obesity (E65–E68); alcoholism and related liver disorders (F10 and K70); tobacco use related disorders (F17, T65.2, Z71.6, Z72.0); depression (F32 and F33); anxiety disorders (F40–F43); hypertension (I10–I15); chronic obstructive pulmonary disease (J40–J47); diabetes mellitus (E10–E14); and hyperlipidemia (E78.0–E78.5).

### Statistical analysis

Person-years were calculated from the start of follow-up until the first hospitalization due to CHD, death, emigration, or the end of the study period (December 31, 2017). The associations between the individual level covariates and CHD were analyzed through Cox regression models. To study the association between the covariates and the time to the first CHD event (incident and fatal) during the study period we used Cox proportional hazard models to estimate Hazard ratios (HRs) with 95% Confidence intervals (CIs). First, we performed a univariate Cox regression for each variable. Thereafter, we conducted a multivariate Cox regression model including all covariates. Interaction tests were also performed in order to examine whether the association between neighborhood deprivation and CHD among patients with bipolar disorder was affected by any of the individual variables. Considering competing risks, further Cox regression using competing risk modelling was conducted, in which death due to other causes was treated as a competing risk for mortality of CHD^25,26^. Furthermore, we found no substantial departures from the proportional hazard assumptions when they were checked by plotting the incidence rates over time and calculating Schoenfeld (partial) residuals. All statistical analyses were performed using SAS 9.4.

### Ethical considerations

This was a nationwide non-intervention register study utilizing already collected and encrypted secondary data. Access to the used national registries was obtained from Swedish authorities prior to the study and all methods were used in accordance with national guidelines and regulations. The permission to take informed consent was formally waived and the study was approved by the Ethical Review Board in Lund, Sweden.

## Results

Table 1 shows the study population comprising a total of 61,114 patients with bipolar disorder, number of incident and fatal CHD events and incidence and mortality rates of CHD by neighborhood-level deprivation. During the follow-up (mean follow-up = 7.7 years), there were 1961 and 2086 incident CHD and 649 and 706 fatal CHD events among the men and women with bipolar disorder, respectively. Population and cumulative incident and mortality of CHD in patients with bipolar disorder are shown in Supplementary Tables S1 and S2, respectively.

**Table 1:** Distribution of population, number of cases, and cumulative rates of incident and fatal CHD of patients with bipolar disorder, 1997–2017.

Neighborhood deprivation	Population		Incident CHD			Fatal CHD		
	No	%	No	%	Rate per 100 individuals	No	%	Rate per 100 individuals
**All**	61,114		4047		6.6	1355		2.2
Low	13,189	21.6	733	18.1	5.6	201	14.8	1.5
Moderate	35,213	57.6	2462	60.8	7.0	819	60.4	2.3
High	12,712	20.8	852	21.1	6.7	335	24.7	2.6
**Men**	24,014		1961		8.2	649		2.7
Low	5219	21.7	379	19.3	7.3	113	17.4	2.2
Moderate	13,905	57.9	1188	60.6	8.5	387	59.6	2.8
**Women**	37,100		2086		11.7	706		1.9
Low	7970	21.5	354	17.0	13.0	88	12.5	1.1
Moderate	21,308	57.4	1274	61.1	10.3	432	61.2	2.0
High	7822	21.1	458	22.0	13.7	186	26.3	2.4

*CHD* Coronary heart disease.

The proportion of patients with bipolar disorder affected with CHD increased among individuals living in high-deprivation neighborhoods. Supplementary Fig. S1 shows the Kaplan–Meir curves for the duration of survival until fatal CHD by different levels of neighborhood deprivation. A graded effect appeared in the beginning of the curves where higher incident and fatal CHD rates were observed when levels of neighborhood deprivation increased (Supplementary Fig. S2).

**Figure 1:**
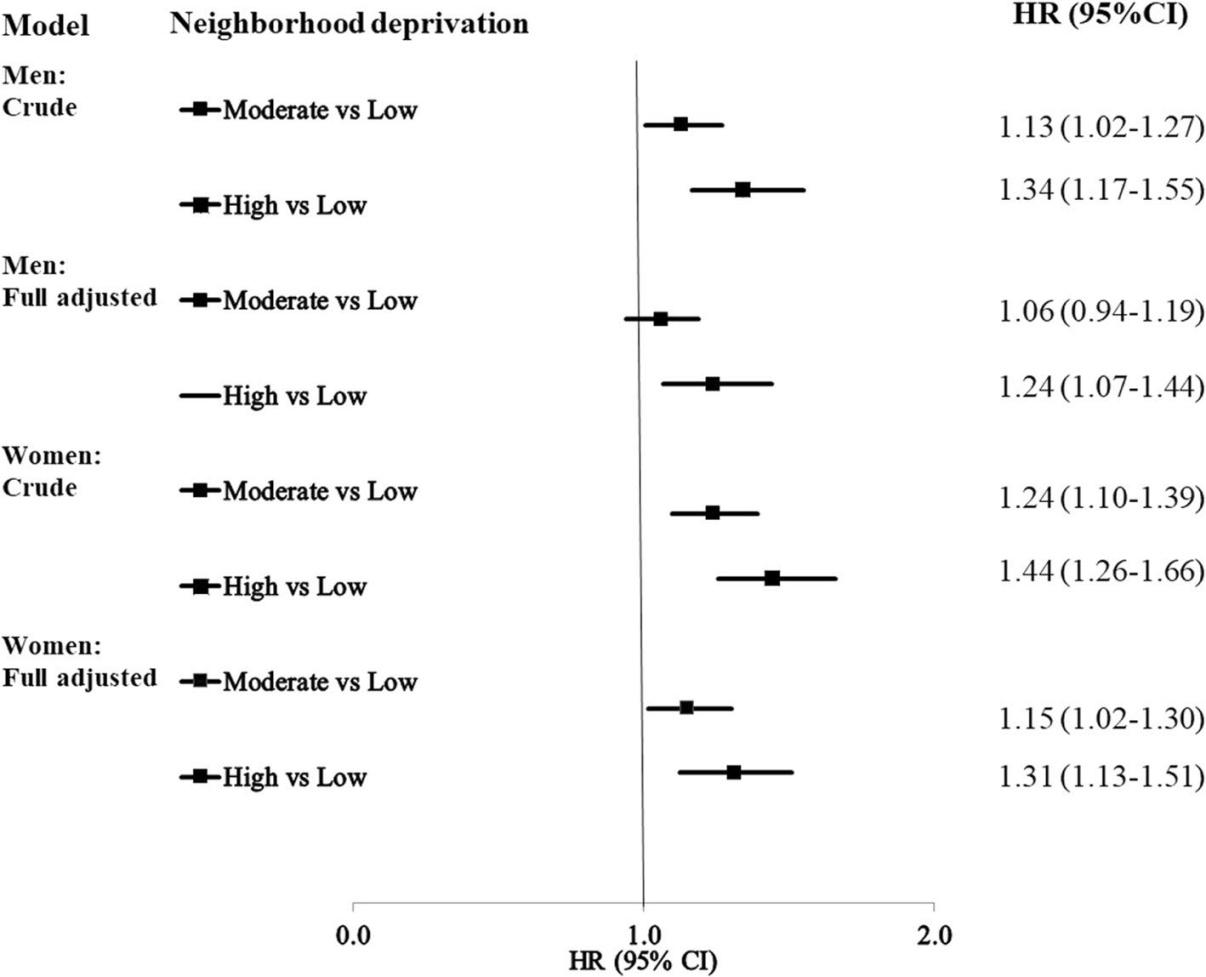
Hazard ratios (HR) and 95% confidence intervals (CI) for incident CHD in patients with bipolar disorder. The full model is adjusted for all the individual level sociodemographic variables and comorbidities. *CHD* Coronary heart disease *HR* Hazard ratio, *CI* Confidence interval.

Figure 1 shows Hazard ratios (HRs) for incident CHD in men and women. The results suggest a gradient, i.e. the CHD incidence became greater with increasing neighborhood deprivation. In the crude model for men, the HRs were 1.13 (95% CI 1.02–1.27) and 1.34 (95% CI 1.17–1.55) in moderate- and high-deprivation neighborhoods, respectively. The HRs decreased after adjustment for the individual-level variables but remained significant in high-deprivation neighborhoods (HR 1.24, 95% CI 1.07–1.44). The corresponding figures of CHD for women were 1.24 (95% CI 1.10–1.39) and 1.44 (95% CI 1.26–1.66) in the crude model. The results of the full model in women show that the HRs decreased, after adjustment for the individual-level variables; the HRs in the full model remained, however, significant in both moderate- (HR 1.15, 95% CI 1.02–1.30) and high-deprivation neighborhoods (HR 1.31, 95% CI 1.13–1.51). The HRs of incident CHD in the different models in men and women are shown in Supplementary Table S3 and Supplementary Table S4. These tables include the results for the individual variables as well.

**Figure 2:**
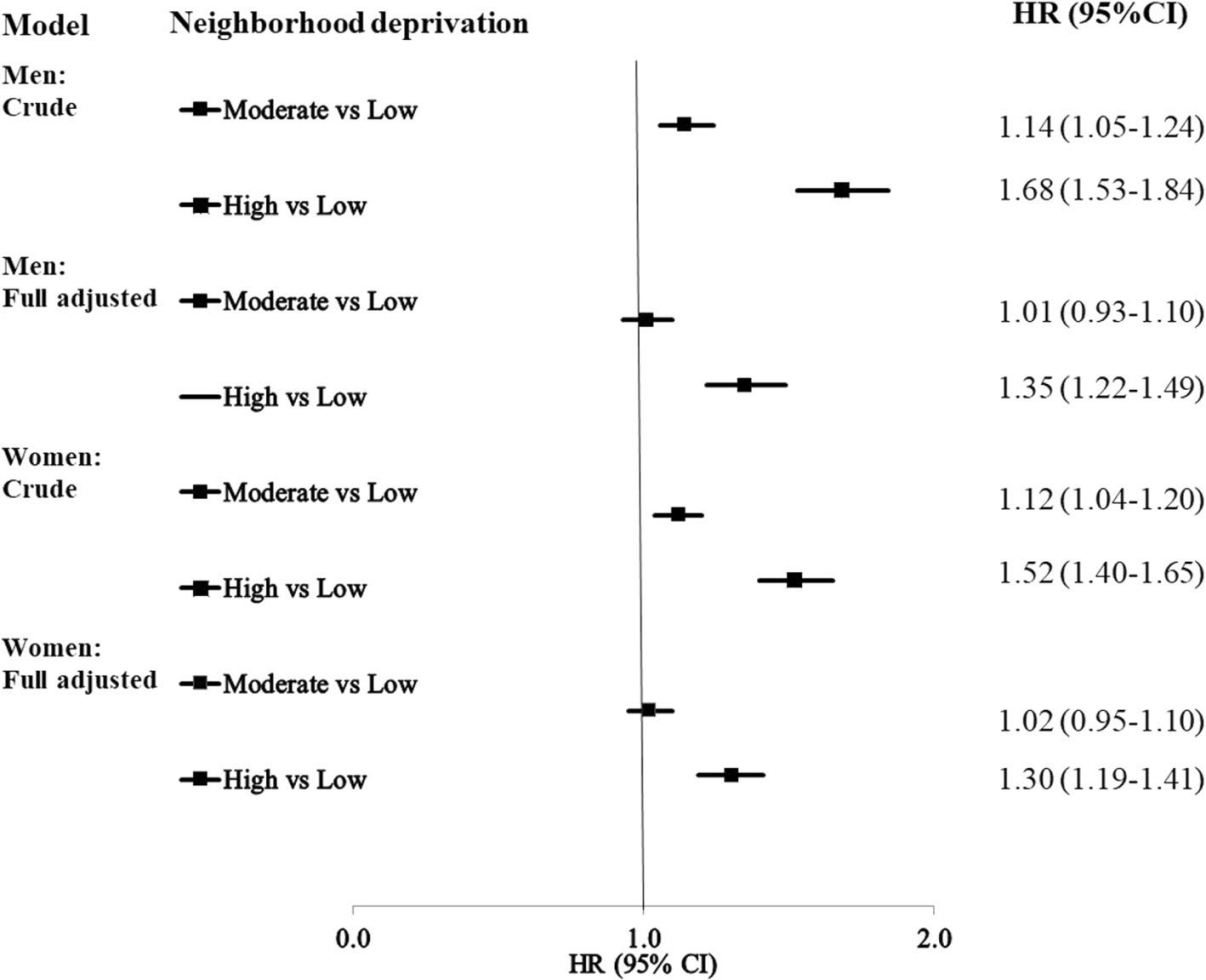
Hazard ratios (HR) and 95% confidence intervals (CI) for fatal CHD in patients with bipolar disorder. The full model is adjusted for all the individual level sociodemographic variables and comorbidities. *CHD* Coronary heart disease, *HR* Hazard ratio, *CI* Confidence interval.

The Hazard ratios (HRs) for fatal CHD in men and women are shown in Fig. 2. The results of the full model are significant in high-deprivation neighborhoods in men (HR 1.35, 95% CI 1.22–1.49) and women (HR 1.30, 95% CI 1.19–1.41), after adjustment for the individual-level variables. The HRs of fatal CHD in the different models in men and women are shown in Supplementary Tables S5 and S6.

Some of the individual-level variables were significantly associated with incident and fatal CHD in the full models. The HRs for CHD were higher for men than women and for those with low education, low family income, a country of birth outside Sweden, or with a hospitalization for comorbidities (Supplementary Tables S7 and S8).

## Discussion

The main finding of this study was that the risk of incident and fatal CHD is higher among patients with bipolar disorder living in deprived neighborhoods than among patients with bipolar disorder living in more affluent neighborhoods. This association was attenuated but remained significant, after adjustment for the individual-level sociodemographic variables and traditional cardiovascular risk factors. Thus, the novel contribution with this study is that it adds evidence on that the incidence rate and fatal rate of CHD increased with the level of neighborhood deprivation in patients with bipolar disorder. Neighborhood deprivation could be recognized as an independent risk factor (approximately 1.3-fold risk) of incident and fatal coronary heart disease in men and women with bipolar disorder.

Systematic reviews have found that diabetes and related factors such as obesity, metabolic syndrome, and lifestyle factors account for a significant amount of the health disparity between people with bipolar disorder and the general population^27–33^. Several cardiovascular risk factors are thus more common in individuals with bipolar disorder than in the general population, which may help to explain the elevated risk of cardiovascular mortality in people with bipolar disorders. Our study shows that this risk seems to be even more pronounced among those exposed to high levels of neighborhood deprivation, which, to the best of our knowledge, has not been studied before.

The causal pathways between neighborhood deprivation and cardiovascular health outcomes are not fully understood^12,17,34,35^ and several possible mechanisms behind our findings are thus plausible. For example, research suggests that diabetes and bipolar disorder co-occurrence could be associated with neighborhood deprivation, but available evidence has been sparse and inconclusive^14^. Another explanation, beyond mechanisms attributed to the bipolar disorder, could be that diabetes and cardiovascular disease risk factors (e.g., tobacco smoking, physical inactivity, and obesity) are more common among individuals living in less prosperous neighborhoods than among those living in more affluent neighborhoods^17^. It is also possible that mechanisms behind our findings may be caused by potential differences between sociodemographic groups (reflected in level of neighborhood deprivation) in attitudes, knowledge, and beliefs that could lead to differences in lifestyle^36^. Although we did not have access to such data in our nationwide study population, such differences might explain parts of the risk differences in CHD across sociodemographic strata in people with bipolar disorders. Furthermore, although Sweden has a universal healthcare system, it is possible that there still are differences between the access to healthcare at the regional and neighborhood level, affecting CHD risk even more among vulnerable individuals, such as those who suffer from bipolar disorder. These differences could be related both to individual socioeconomic differences that may affect people’s possibilities to redeem medical prescriptions^37^ and the lower access to primary healthcare seen in less affluent neighborhoods^38^.

Other potential mechanisms behind our findings may be external. For example, the crime levels are often higher in more deprived neighborhoods^39^, which could increase psychosocial stress and reduce outdoor physical activity due to fear of going outside. Social capital has also been found to be lower in less affluent neighborhoods, which in turn is related to social norms, beliefs and attitudes^40^. Others have also hypothesized that persons living in deprived neighborhoods might be at higher risk of unfavorable societal stressors (e.g., air pollution, noise, and violence)^41^, which may culminate in chronic psychological stress and predispose individuals to adverse health outcomes, including CHD. Furthermore, it has, previously and elsewhere, been suggested that health-promoting neighborhood goods, services, and resources are lower in less affluent neighborhoods. However, this does not seem to be the case in Sweden, as we have previously shown that the availability of potentially health-promoting goods, services, and resources actually seems to be higher in deprived neighborhoods^42^. Therefore, inequalities of access to healthcare is probably not a possible mechanism, although the utilization of healthcare might be different between population subgroups. Although the availability of potentially health-promoting goods, services, and resources are higher in deprived neighborhoods, this is also the case for health-damaging neighborhood features^42^, which may partly explain the negative effects on CHD of living in a deprived neighborhood.

Additional findings in the present study were that women with bipolar disorder seemed to be more affected by neighborhood deprivation than men with bipolar disorder in regard to incident CHD. These findings were in-line with an earlier study of the general population^12^ on the association between neighborhood deprivation and incident CHD in Sweden. In general, women may spend more time in their immediate neighborhood than men (although men have a higher absolute risk of CHD than women). However, the findings were reversed for fatal CHD, which could have several explanations. For example, a different distribution of comorbidities and/or healthcare seeking patterns may exist between men and women. Moreover, contrasting findings on the associations with incident and fatal CHD were also observed for certain individual-level sociodemographic factors (e.g. family income) and comorbidities (e.g. anxiety, depression, and obesity), which also could be explained, *inter alia*, by differences in healthcare seeking patterns. For example, it is possible that individuals with bipolar disorder and low income and/or certain comorbidities would be more (or less) prone to seek healthcare for CHD symptoms, which could affect the rates of incident and fatal CHD differently. Further studies are, however, needed to confirm our findings in other settings and to examine the potential mechanisms behind the discrepancies between CHD incidence and mortality in the observed associations.

This present study has some important limitations. Most notably, we had no data on several risk factors for CHD, such as smoking, high-calorie diet, or physical inactivity. However, some prior works on socioeconomic status and CHD risk have adjusted for physical inactivity as well as tobacco smoking and still found an independent association^41,43^. We also had no data on bipolar disorder related medications, such as atypical antipsychotics, associated to high risk of cardiometabolic risk factors^30,44^. Moreover, mobility (i.e. moving between neighborhoods of different level of deprivation) could affect the results in studies exploring neighborhood deprivation and health. However, we did not adjust for mobility as only a few events (i.e. 3 incident- and 0 fatal CHD events) were observed in the individuals in the present study population who had moved during the study period. Finally, we had no data on quality of healthcare in the neighborhood; thus, we could not assess if this was an important mechanism behind our findings. Nevertheless, the limitations are balanced out by the several strengths. Firstly, the cohort was of considerable size as it included practically all patients with bipolar disorder (≥ 30 years of age) in Sweden during the study period, which increases the generalizability of our results. Another strength was the personal identification number (encrypted in this study), assigned to each individual in Sweden, which gave us the opportunity to follow the participants with more or less no loss to follow-up. Thirdly, the outcome data were based on clinical diagnoses, registered by physicians, rather than self-reported data, which eliminated any recall bias. An additional key strength was the access to SAMS units’ data. These units defined small geographic boundaries of neighborhoods with relatively homogenous types of buildings and included about 1000–2000 persons within each unit. The small size of the units were a strength as small neighborhoods have previously been shown to correspond well with how the residents define their neighborhoods^45^. Moreover, our data were highly complete and only 0.6% of the study participants were excluded because of missing SAMS codes. The national demographic and individual sociodemographic data were also highly complete—less than 1% of the data were missing. We were thus able to link clinical data from individual patients to comprehensive national demographic and socioeconomic data. Lastly, the findings were in line with previous findings on neighborhood deprivation and health in general, including previous evidence on the association between neighborhood deprivation in relation to cardiovascular risk factors^9–11,13,14^ and coronary heath disease^12^.

In conclusion, this nationwide cohort study showed that, after accounting for individual-level sociodemographic factors, neighborhood deprivation was independently associated with (increased risk of) incident and fatal CHD among patients with bipolar disorder. These findings are useful for healthcare workers encountering patients with bipolar disorder and particularly those living in deprived neighborhoods. Healthcare planners and policy makers could use this new knowledge to focus extra attention on cardiovascular disease prevention in patients suffering from bipolar disorders and living in deprived neighborhoods. Understanding the pathways between neighborhood factors (independent of individual factors) and various health outcomes in patients with bipolar disorder is challenging and could be further examined in smaller, qualitative studies.

## Supplementary Information

Supplementary Information.

## Data Availability

This study made use of several national registers and, owing to legal concerns, data cannot be made openly available. Further information regarding the health registries is available from the Swedish National Board of Health and Welfare (https://www.socialstyrelsen.se/en/statistics-and-data/registers/) and *Statistics Sweden *(https://www.scb.se/en/). The code used in the analysis can be provided upon reasonable request directed to Professor Kristina Sundquist.

## Abbreviations

CHD: Coronary heart disease
CI: Confidence interval
HR: Hazard ratio
ICD: International classification of diseases
SAMS: Small area market statistics
