# Supplementary material for: Neighborhood deprivation and coronary heart disease in patients with bipolar disorder
Source: Sci Rep. 2022 Oct 6;12:16763. doi: 10.1038/s41598-022-21295-0 (PMC9537303; doi:10.1038/s41598-022-21295-0)
Supplement: Supplementary file 1 — Supplementary Information. [file 41598_2022_21295_MOESM1_ESM.doc]

**Title**: Neighborhood deprivation and coronary heart disease in patients with bipolar disorder

**Authors:** Filip Jansåker ^a,b*^ MD, PhD; Veronica Milos Nymberg ^a^ MD, PhD; Jan Sundquist ^a,c,d^ MD, PhD; Kenta Okuyama ^a^ PhD-student; Tsuyoshi Hamano ^d,e^ PhD; Kristina Sundquist ^a,b,c^ MD, PhD; Xinjun Li ^a^ MD, PhD.

**Supplemental online content:**

**Figure legends**

**Supplementary Figure S1.** The Kaplan–Meier curves for the duration of survival of coronary heart disease in patients with bipolar disorder.

**Supplementary Figure S2.** Cumulative rate (per 100 individuals) of incident and fatal coronary heart disease (CHD) in patients with bipolar disorder by different levels of neighborhood deprivation.

**Table legends**

**Supplementary Table S1**. Distribution of population, number of cases, and cumulative rates of incident CHD in patients with bipolar disorder, 1997-2017

**Supplementary Table S2**. Distribution of population, number of cases, and cumulative rates of fatal CHD in patients with bipolar disorder, 1997-2017

**Supplementary Table S3.** Hazard ratios (HR) and 95% confidence intervals (CI) for incident CHD in men; Results of Cox regression models

**Supplementary Table S4.** Hazard ratios (HR) and 95% confidence intervals (CI) for incident CHD in women; Results of Cox regression models

**Supplementary Table S5.** Hazard ratios (HR) and 95% confidence intervals (CI) for fatal CHD in men; Results of multivariable competing risk survival analysis

**Supplementary Table S6.** Hazard ratios (HR) and 95% confidence intervals (CI) for fatal CHD in women; Results of multivariable competing risk survival analysis

**Supplementary Table S7.** Hazard ratios (HR) and 95% confidence intervals (CI) for incident CHD; Results of Cox regression models

**Supplementary Table S8.** Hazard ratios (HR) and 95% confidence intervals (CI) for fatal CHD; Results of multivariable competing risk survival analysis

**Supplementary Figure S1.** The Kaplan–Meier curves for the duration of survival of coronary heart disease in patients with bipolar disorder.

P-value <.001

**Supplementary Figure S2.** Cumulative rate (per 100 individuals) of incident and fatal coronary heart disease

(CHD) in patients with bipolar disorder by different levels of neighborhood deprivation.

**
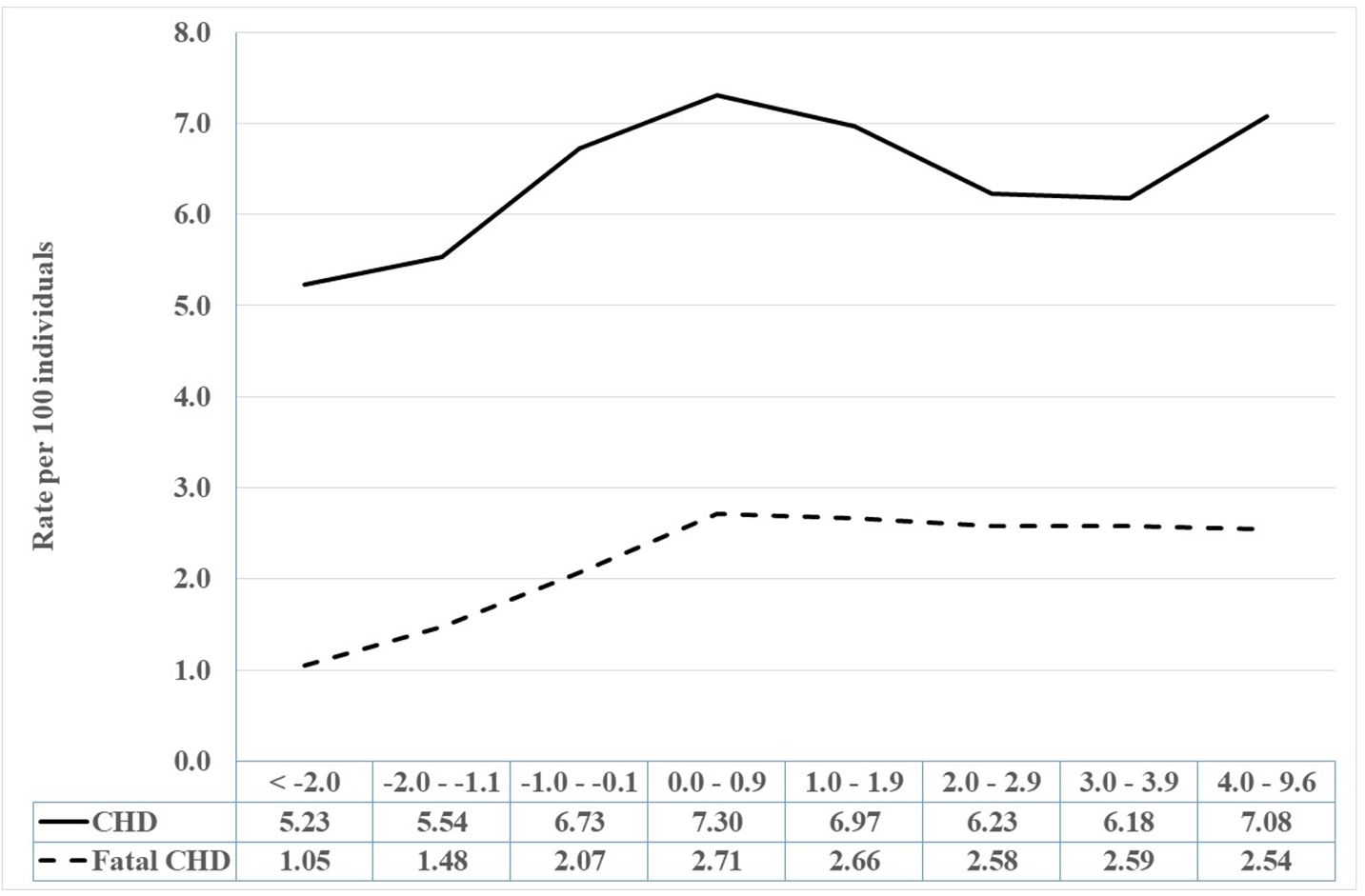
**

| **Supplementary Table S1.** Distribution of population, number of cases, and cumulative rates of incident CHD in patients with bipolar disorder, 1997-2017 | | | | | | | | | | |
| --- | --- | --- | --- | --- | --- | --- | --- | --- | --- | --- |
|  | Population | | |  | Incident CHD | |  | Cumulative rates (%) of CHD by neighborhood deprivation | | |
|  | No. | | % |  | No. | % |  | Low (n=13189) | Moderate (n=35213) | High (n=12712) |
| Total population | 61114 | |  |  | 4047 |  |  | 5.6 | 7.0 | 6.7 |
| **Gender** |  | |  |  |  |  |  |  |  |  |
| Men | 24014 | | 39.3 |  | 1961 | 48.5 |  | 7.3 | 8.5 | 8.1 |
| Women | 37100 | | 60.7 |  | 2086 | 51.5 |  | 4.4 | 6.0 | 5.9 |
| **Age (years)** |  | |  |  |  |  |  |  |  |  |
| 30-49 | 32126 | | 52.6 |  | 813 | 20.1 |  | 1.8 | 2.7 | 2.9 |
| 50-59 | 13359 | | 21.9 |  | 1106 | 27.3 |  | 6.5 | 8.7 | 9.0 |
| 60-69 | 8689 | | 14.2 |  | 1079 | 26.7 |  | 10.9 | 12.6 | 13.5 |
| 70-79 | 4618 | | 7.6 |  | 716 | 17.7 |  | 14.1 | 16.3 | 14.5 |
| ≥ 80 | 2322 | | 3.8 |  | 333 | 8.2 |  | 14.2 | 15.2 | 12.1 |
| **Education attainment** |  | |  |  |  |  |  |  |  |  |
| ≤ 9 years | 13677 | | 22.4 |  | 1335 | 33.0 |  | 9.8 | 10.4 | 8.4 |
| 10–11 years | 17985 | | 29.4 |  | 1289 | 31.9 |  | 6.3 | 7.3 | 7.4 |
| ≥ 12 years | 29452 | | 48.2 |  | 1423 | 35.2 |  | 4.4 | 5.1 | 4.9 |
| **Family income** |  | |  |  |  |  |  |  |  |  |
| Low income | 15257 | | 25.0 |  | 1093 | 27.0 |  | 6.1 | 7.4 | 7.3 |
| Middle-low income | 15280 | | 25.0 |  | 1046 | 25.8 |  | 5.7 | 7.2 | 6.8 |
| Middle-high income | 15285 | | 25.0 |  | 1047 | 25.9 |  | 5.7 | 7.4 | 6.5 |
| High income | 15292 | | 25.0 |  | 861 | 21.3 |  | 5.1 | 5.9 | 5.6 |
| **Region of residence** |  | |  |  |  |  |  |  |  |  |
| Large cities | 30907 | | 50.6 |  | 1887 | 46.6 |  | 5.1 | 6.5 | 6.3 |
| Middle-sized towns | 19550 | | 32.0 |  | 1197 | 29.6 |  | 5.2 | 6.3 | 6.2 |
| Small towns/rural areas | 10657 | | 17.4 |  | 963 | 23.8 |  | 7.9 | 9.2 | 9.5 |
| **Marital status** |  | |  |  |  |  |  |  |  |  |
| Married/cohabiting | 21273 | | 34.8 |  | 1653 | 40.8 |  | 6.6 | 8.2 | 8.2 |
| Not married | 39841 | | 65.2 |  | 2394 | 59.2 |  | 4.8 | 6.4 | 6.1 |
| **Immigrant status** |  | |  |  |  |  |  |  |  |  |
| Born in Sweden | 52546 | | 86.0 |  | 3526 | 87.1 |  | 5.6 | 7.1 | 6.8 |
| Born in other countries | 8568 | | 14.0 |  | 521 | 12.9 |  | 4.9 | 6.3 | 6.4 |
| **Hospitalization of COPD** |  | |  |  |  |  |  |  |  |  |
| No | 57761 | | 94.5 |  | 3827 | 94.6 |  | 5.5 | 7.0 | 6.8 |
| Yes | 3353 | | 5.5 |  | 220 | 5.4 |  | 6.9 | 7.0 | 5.3 |
| **Hospitalization of alcoholism and related liver disorders** | |  |  |  |  |  |  |  |  |  |
| No | 56245 | | 92.0 |  | 3502 | 86.5 |  | 5.2 | 6.6 | 6.2 |
| Yes | 4869 | | 8.0 |  | 545 | 13.5 |  | 9.9 | 11.3 | 11.9 |
| **Hospitalization of obesity** |  | |  |  |  |  |  |  |  |  |
| No | 50972 | | 83.4 |  | 3462 | 85.5 |  | 5.6 | 7.2 | 6.9 |
| Yes | 10142 | | 16.6 |  | 585 | 14.5 |  | 5.3 | 5.9 | 5.9 |
| **Hospitalization of depression** |  | |  |  |  |  |  |  |  |  |
| No | 29918 | | 49.0 |  | 2228 | 55.1 |  | 6.2 | 8.0 | 7.2 |
| Yes | 31196 | | 51.0 |  | 1819 | 44.9 |  | 4.9 | 6.0 | 6.2 |
| **Hospitalization of anxiety** |  | |  |  |  |  |  |  |  |  |
| No | 33792 | | 55.3 |  | 2724 | 67.3 |  | 6.6 | 8.6 | 8.1 |
| Yes | 27322 | | 44.7 |  | 1323 | 32.7 |  | 4.1 | 4.9 | 5.2 |
| **Hospitalization of hypertension** |  | |  |  |  |  |  |  |  |  |
| No | 53385 | | 87.4 |  | 2613 | 64.6 |  | 4.0 | 5.2 | 5.1 |
| Yes | 7729 | | 12.6 |  | 1434 | 35.4 |  | 17.1 | 19.3 | 17.8 |
| **Hospitalization of diabetes** |  | |  |  |  |  |  |  |  |  |
| No | 57761 | | 94.5 |  | 3827 | 94.6 |  | 5.5 | 7.0 | 6.8 |
| Yes | 3353 | | 5.5 |  | 220 | 5.4 |  | 6.9 | 7.0 | 5.3 |
| **Hospitalization of hyperlipidemia** |  | |  |  |  |  |  |  |  |  |
| No | 55487 | | 90.8 |  | 3128 | 77.3 |  | 4.7 | 6.0 | 5.5 |
| Yes | 5627 | | 9.2 |  | 919 | 22.7 |  | 16.4 | 16.2 | 16.5 |
| **Hospitalization of tobacco use disorder** |  | |  |  |  |  |  |  |  |  |
| No | 57761 | | 94.5 |  | 3827 | 94.6 |  | 5.5 | 7.0 | 6.8 |
| Yes | 3353 | | 5.5 |  | 220 | 5.4 |  | 6.9 | 7.0 | 5.3 |

CHD: Coronary heart disease; COPD: Chronic obstructive pulmonary disease.

| **Supplementary Table S2.** Distribution of population, number of cases, and cumulative rates of fatal CHD in patients with bipolar disorder, 1997-2017 | | | | | | | | | | |
| --- | --- | --- | --- | --- | --- | --- | --- | --- | --- | --- |
|  |  | |  |  | Fatal CHD | |  | Cumulative rates (%) of fatal CHD by neighborhood deprivation | | |
|  |  | |  |  | No. | % |  | Low (n=13189) | Moderate (n=35213) | High (n=12712) |
| Total population | 61114 | |  |  | 1355 |  |  | 1.5 | 2.3 | 2.6 |
| **Gender** |  | |  |  |  |  |  |  |  |  |
| Men | 24014 | | 39.3 |  | 649 | 47.9 |  | 2.2 | 2.8 | 3.0 |
| Women | 37100 | | 60.7 |  | 706 | 52.1 |  | 1.1 | 2.0 | 2.4 |
| **Age (years)** |  | |  |  |  |  |  |  |  |  |
| 30-49 | 32126 | | 52.6 |  | 127 | 9.4 |  | 0.2 | 0.4 | 0.5 |
| 50-59 | 13359 | | 21.9 |  | 296 | 21.8 |  | 1.6 | 2.2 | 3.0 |
| 60-69 | 8689 | | 14.2 |  | 366 | 27.0 |  | 2.1 | 4.2 | 6.6 |
| 70-79 | 4618 | | 7.6 |  | 332 | 24.5 |  | 5.7 | 7.8 | 6.9 |
| ≥ 80 | 2322 | | 3.8 |  | 234 | 17.3 |  | 8.8 | 10.6 | 9.8 |
| **Education attainment** |  | |  |  |  |  |  |  |  |  |
| ≤ 9 years | 13677 | | 22.4 |  | 636 | 46.9 |  | 3.5 | 5.0 | 4.5 |
| 10–11 years | 17985 | | 29.4 |  | 340 | 25.1 |  | 1.4 | 1.9 | 2.2 |
| ≥ 12 years | 29452 | | 48.2 |  | 379 | 28.0 |  | 1.2 | 1.3 | 1.5 |
| **Family income** |  | |  |  |  |  |  |  |  |  |
| Low income | 15257 | | 25.0 |  | 471 | 34.8 |  | 2.1 | 3.1 | 3.5 |
| Middle-low income | 15280 | | 25.0 |  | 412 | 30.4 |  | 2.1 | 2.8 | 2.9 |
| Middle-high income | 15285 | | 25.0 |  | 319 | 23.5 |  | 1.5 | 2.3 | 2.2 |
| High income | 15292 | | 25.0 |  | 153 | 11.3 |  | 1.0 | 1.0 | 1.0 |
| **Region of residence** |  | |  |  |  |  |  |  |  |  |
| Large cities | 30907 | | 50.6 |  | 715 | 52.8 |  | 1.5 | 2.5 | 2.9 |
| Middle-sized towns | 19550 | | 32.0 |  | 432 | 31.9 |  | 1.3 | 2.3 | 2.6 |
| Small towns/rural areas | 10657 | | 17.4 |  | 208 | 15.4 |  | 1.8 | 2.0 | 1.8 |
| **Marital status** |  | |  |  |  |  |  |  |  |  |
| Married/cohabiting | 21273 | | 34.8 |  | 432 | 31.9 |  | 1.4 | 2.1 | 2.8 |
| Not married | 39841 | | 65.2 |  | 923 | 68.1 |  | 1.7 | 2.4 | 2.6 |
| **Immigrant status** |  | |  |  |  |  |  |  |  |  |
| Born in Sweden | 52546 | | 86.0 |  | 1220 | 90.0 |  | 1.6 | 2.4 | 3.0 |
| Born in other countries | 8568 | | 14.0 |  | 135 | 10.0 |  | 1.1 | 1.9 | 1.4 |
| **Hospitalization of COPD** |  | |  |  |  |  |  |  |  |  |
| No | 57761 | | 94.5 |  | 1320 | 97.4 |  | 1.6 | 2.4 | 2.7 |
| Yes | 3353 | | 5.5 |  | 35 | 2.6 |  | 0.4 | 1.2 | 1.1 |
| **Hospitalization of alcoholism and related liver disorders** | |  |  |  |  |  |  |  |  |  |
| No | 56245 | | 92.0 |  | 1168 | 86.2 |  | 1.4 | 2.2 | 2.5 |
| Yes | 4869 | | 8.0 |  | 187 | 13.8 |  | 2.8 | 4.2 | 3.9 |
| **Hospitalization of obesity** |  | |  |  |  |  |  |  |  |  |
| No | 50972 | | 83.4 |  | 1162 | 85.8 |  | 1.5 | 2.4 | 2.7 |
| Yes | 10142 | | 16.6 |  | 193 | 14.2 |  | 1.5 | 1.9 | 2.3 |
| **Hospitalization of depression** |  | |  |  |  |  |  |  |  |  |
| No | 29918 | | 49.0 |  | 870 | 64.2 |  | 2.0 | 3.1 | 3.4 |
| Yes | 31196 | | 51.0 |  | 485 | 35.8 |  | 1.1 | 1.6 | 1.9 |
| **Hospitalization of anxiety** |  | |  |  |  |  |  |  |  |  |
| No | 33792 | | 55.3 |  | 1055 | 77.9 |  | 2.1 | 3.3 | 3.9 |
| Yes | 27322 | | 44.7 |  | 300 | 22.1 |  | 0.8 | 1.1 | 1.3 |
| **Hospitalization of hypertension** |  | |  |  |  |  |  |  |  |  |
| No | 53385 | | 87.4 |  | 1054 | 77.8 |  | 1.3 | 2.1 | 2.3 |
| Yes | 7729 | | 12.6 |  | 301 | 22.2 |  | 2.8 | 3.9 | 5.0 |
| **Hospitalization of diabetes** |  | |  |  |  |  |  |  |  |  |
| No | 57761 | | 94.5 |  | 1320 | 97.4 |  | 1.6 | 2.4 | 2.7 |
| Yes | 3353 | | 5.5 |  | 35 | 2.6 |  | 0.4 | 1.2 | 1.1 |
| **Hospitalization of hyperlipidemia** |  | |  |  |  |  |  |  |  |  |
| No | 55487 | | 90.8 |  | 1081 | 79.8 |  | 1.4 | 2.1 | 2.3 |
| Yes | 5627 | | 9.2 |  | 274 | 20.2 |  | 3.7 | 5.0 | 5.3 |
| **Hospitalization of tobacco use disorder** |  | |  |  |  |  |  |  |  |  |
| No | 57761 | | 94.5 |  | 1320 | 97.4 |  | 1.6 | 2.4 | 2.7 |
| Yes | 3353 | | 5.5 |  | 35 | 2.6 |  | 0.4 | 1.2 | 1.1 |

CHD: Coronary heart disease; COPD: Chronic obstructive pulmonary disease.

| **Supplementary Table S3.** Hazard ratios (HR) and 95% confidence intervals (CI) for incident CHD in men; Results of Cox regression models | | | | | | | | | | | |  |
| --- | --- | --- | --- | --- | --- | --- | --- | --- | --- | --- | --- | --- |
|  | Model 1 | | |  | Model 2 | | |  | Model 3 | | |  |
|  | HR | 95% CI | |  | HR | 95% CI | |  | HR | 95% CI | | P-value |
| **Neighborhood deprivation** (ref. Low) |  |  |  |  |  |  |  |  |  |  |  |  |
| Moderate | 1.13 | 1.01 | 1.27 |  | 1.07 | 0.95 | 1.21 |  | 1.06 | 0.94 | 1.19 | 0.3477 |
| High | 1.34 | 1.17 | 1.55 |  | 1.30 | 1.12 | 1.50 |  | 1.24 | 1.07 | 1.44 | 0.0040 |
| Age | 1.07 | 1.06 | 1.07 |  | 1.06 | 1.06 | 1.07 |  | 1.06 | 1.06 | 1.07 | <.0001 |
| Family income (ref. Highest quartiles) |  |  |  |  |  |  |  |  |  |  |  |  |
| Low |  |  |  |  | 0.89 | 0.78 | 1.02 |  | 0.92 | 0.80 | 1.05 | 0.2124 |
| Middle-low |  |  |  |  | 0.76 | 0.67 | 0.87 |  | 0.74 | 0.64 | 0.84 | <.0001 |
| Middle-high |  |  |  |  | 1.04 | 0.92 | 1.17 |  | 1.00 | 0.89 | 1.13 | 0.9741 |
| Education attainment (ref. ≥ 12 years) |  |  |  |  |  |  |  |  |  |  |  |  |
| ≤ 9 years |  |  |  |  | 1.24 | 1.11 | 1.39 |  | 1.23 | 1.10 | 1.38 | 0.0003 |
| 10–11 years |  |  |  |  | 1.23 | 1.10 | 1.37 |  | 1.19 | 1.07 | 1.33 | 0.0018 |
| Country of origin (ref. Sweden) |  |  |  |  | 1.23 | 1.07 | 1.41 |  | 1.22 | 1.06 | 1.41 | 0.0046 |
| Marital status (ref. Married/cohabiting) |  |  |  |  | 0.99 | 0.90 | 1.09 |  | 1.01 | 0.91 | 1.11 | 0.9229 |
| Region of residence (ref. Large cities) |  |  |  |  |  |  |  |  |  |  |  |  |
| Middle-sized towns |  |  |  |  | 0.94 | 0.85 | 1.04 |  | 0.95 | 0.86 | 1.06 | 0.3366 |
| Small towns/rural areas |  |  |  |  | 1.52 | 1.35 | 1.70 |  | 1.52 | 1.36 | 1.71 | <.0001 |
| Hospitalization for obesity (ref. Non) |  |  |  |  |  |  |  |  | 1.41 | 1.13 | 1.76 | 0.0021 |
| Hospitalization for COPD (ref. Non) |  |  |  |  |  |  |  |  | 1.35 | 1.17 | 1.56 | <.0001 |
| Hospitalization for alcoholism and related liver disorders (ref. Non) |  |  |  |  |  |  |  |  | 1.02 | 0.91 | 1.15 | 0.7321 |
| Hospitalization for depression (ref. Non) |  |  |  |  |  |  |  |  | 0.96 | 0.88 | 1.06 | 0.4262 |
| Hospitalization for anxiety (ref. Non) |  |  |  |  |  |  |  |  | 1.09 | 0.98 | 1.22 | 0.0981 |
| Hospitalization for hypertension (ref. Non) |  |  |  |  |  |  |  |  | 1.95 | 1.77 | 2.15 | <.0001 |
| Hospitalization for diabetes (ref. Non) |  |  |  |  |  |  |  |  | 1.61 | 1.45 | 1.79 | <.0001 |
| Hospitalization for hyperlipidemia (ref. Non) |  |  |  |  |  |  |  |  | 2.20 | 1.71 | 2.84 | <.0001 |
| Hospitalization for tobacco use disorder (ref. Non) |  |  |  |  |  |  |  |  | 2.22 | 1.76 | 2.79 | <.0001 |
| HR: Hazard ratio; CI: Confidence interval; CHD: Coronary heart disease; COPD: Chronic obstructive pulmonary disease. | | | | | | | | | | | | |
| Model 1: Univariate model, adjusted for age; Model 2: Adjusted for individual characteristics; Model 3: Full adjusted model. | | | | | | | | | | | | |

| **Supplementary Table S4.** Hazard ratios (HR) and 95% confidence intervals (CI) for incident CHD in women; Results of Cox regression models | | | | | | | | | | | |  |
| --- | --- | --- | --- | --- | --- | --- | --- | --- | --- | --- | --- | --- |
|  | Model 1 | | |  | Model 2 | | |  | Model 3 | | |  |
|  | HR | 95% CI | |  | HR | 95% CI | |  | HR | 95% CI | | P-value |
| **Neighborhood deprivation** (ref. Low) |  |  |  |  |  |  |  |  |  |  |  |  |
| Moderate | 1.24 | 1.10 | 1.39 |  | 1.18 | 1.04 | 1.33 |  | 1.15 | 1.02 | 1.30 | 0.0212 |
| High | 1.44 | 1.26 | 1.66 |  | 1.39 | 1.21 | 1.61 |  | 1.31 | 1.13 | 1.51 | 0.0003 |
| Age | 1.07 | 1.06 | 1.07 |  | 1.07 | 1.06 | 1.07 |  | 1.06 | 1.06 | 1.07 | <.0001 |
| Family income (ref. Highest quartiles) |  |  |  |  |  |  |  |  |  |  |  |  |
| Low |  |  |  |  | 0.85 | 0.74 | 0.98 |  | 0.87 | 0.76 | 1.01 | 0.0593 |
| Middle-low |  |  |  |  | 0.88 | 0.77 | 1.02 |  | 0.88 | 0.76 | 1.01 | 0.0668 |
| Middle-high |  |  |  |  | 0.97 | 0.84 | 1.12 |  | 0.94 | 0.81 | 1.08 | 0.3906 |
| Education attainment (ref. ≥ 12 years) |  |  |  |  |  |  |  |  |  |  |  |  |
| ≤ 9 years |  |  |  |  | 1.25 | 1.11 | 1.41 |  | 1.16 | 1.03 | 1.30 | 0.0153 |
| 10–11 years |  |  |  |  | 1.24 | 1.11 | 1.39 |  | 1.15 | 1.03 | 1.28 | 0.0134 |
| Country of origin (ref. Sweden) |  |  |  |  | 1.16 | 1.02 | 1.32 |  | 1.14 | 1.01 | 1.30 | 0.0404 |
| Marital status (ref. Married/cohabiting) |  |  |  |  | 0.97 | 0.89 | 1.06 |  | 0.99 | 0.90 | 1.08 | 0.8200 |
| Region of residence (ref. Large cities) |  |  |  |  |  |  |  |  |  |  |  |  |
| Middle-sized towns |  |  |  |  | 0.92 | 0.83 | 1.02 |  | 0.92 | 0.83 | 1.02 | 0.0940 |
| Small towns/rural areas |  |  |  |  | 1.79 | 1.61 | 2.00 |  | 1.84 | 1.65 | 2.05 | <.0001 |
| Hospitalization for obesity (ref. Non) |  |  |  |  |  |  |  |  | 1.24 | 1.04 | 1.49 | 0.0193 |
| Hospitalization for COPD (ref. Non) |  |  |  |  |  |  |  |  | 1.51 | 1.34 | 1.70 | <.0001 |
| Hospitalization for alcoholism and related liver disorders (ref. Non) |  |  |  |  |  |  |  |  | 1.08 | 0.93 | 1.26 | 0.3185 |
| Hospitalization for depression (ref. Non) |  |  |  |  |  |  |  |  | 1.01 | 0.92 | 1.10 | 0.9055 |
| Hospitalization for anxiety (ref. Non) |  |  |  |  |  |  |  |  | 1.10 | 0.99 | 1.21 | 0.0707 |
| Hospitalization for hypertension (ref. Non) |  |  |  |  |  |  |  |  | 1.99 | 1.81 | 2.18 | <.0001 |
| Hospitalization for diabetes (ref. Non) |  |  |  |  |  |  |  |  | 1.59 | 1.43 | 1.78 | <.0001 |
| Hospitalization for hyperlipidemia (ref. Non) |  |  |  |  |  |  |  |  | 1.85 | 1.44 | 2.39 | <.0001 |
| Hospitalization for tobacco use disorder (ref. Non) |  |  |  |  |  |  |  |  | 2.16 | 1.74 | 2.70 | <.0001 |
| HR: Hazard ratio; CI: Confidence interval; CHD: Coronary heart disease; COPD: Chronic obstructive pulmonary disease. | | | | | | | | | | | | |
| Model 1: Univariate model, adjusted for age; Model 2: Adjusted for individual characteristics; Model 3: Full model. | | | | | | | | | | | | |

| **Supplementary Table S5.** Hazards ratios (HR) and 95% confidence intervals (CI) for fatal CHD in men; Results of multivariable competing risk survival analysis | | | | | | | | | | | | |  |
| --- | --- | --- | --- | --- | --- | --- | --- | --- | --- | --- | --- | --- | --- |
|  | Model 1 | | |  | Model 2 | | |  | Model 3 | | |  | |
|  | HR | 95% CI | |  | HR | 95% CI | |  | HR | 95% CI | | P-value | |
| **Neighborhood deprivation** (ref. Low) |  |  |  |  |  |  |  |  |  |  |  |  | |
| Moderate | 1.14 | 1.06 | 1.24 |  | 1.00 | 0.92 | 1.09 |  | 1.01 | 0.93 | 1.10 | 0.7704 | |
| High | 1.68 | 1.53 | 1.84 |  | 1.37 | 1.24 | 1.51 |  | 1.35 | 1.22 | 1.49 | <.0001 | |
| Age | 1.08 | 1.07 | 1.08 |  | 1.08 | 1.07 | 1.08 |  | 1.08 | 1.08 | 1.08 | <.0001 | |
| Family income (ref. Highest quartiles) |  |  |  |  |  |  |  |  |  |  |  |  | |
| Low |  |  |  |  | 1.81 | 1.65 | 1.99 |  | 1.74 | 1.59 | 1.91 | <.0001 | |
| Middle-low |  |  |  |  | 1.49 | 1.36 | 1.63 |  | 1.45 | 1.32 | 1.60 | <.0001 | |
| Middle-high |  |  |  |  | 1.33 | 1.21 | 1.46 |  | 1.30 | 1.19 | 1.43 | <.0001 | |
| Education attainment (ref. ≥ 12 years) |  |  |  |  |  |  |  |  |  |  |  |  | |
| ≤ 9 years |  |  |  |  | 1.40 | 1.30 | 1.51 |  | 1.38 | 1.28 | 1.48 | <.0001 | |
| 10–11 years |  |  |  |  | 1.11 | 1.02 | 1.20 |  | 1.11 | 1.02 | 1.20 | 0.0147 | |
| Country of origin (ref. Sweden) |  |  |  |  | 0.84 | 0.76 | 0.93 |  | 0.86 | 0.78 | 0.96 | 0.0047 | |
| Marital status (ref. Married/cohabiting) |  |  |  |  | 1.33 | 1.25 | 1.42 |  | 1.27 | 1.19 | 1.35 | <.0001 | |
| Region of residence (ref. Large cities) |  |  |  |  |  |  |  |  |  |  |  |  | |
| Middle-sized towns |  |  |  |  | 0.84 | 0.79 | 0.90 |  | 0.86 | 0.80 | 0.92 | <.0001 | |
| Small towns/rural areas |  |  |  |  | 0.86 | 0.78 | 0.94 |  | 0.88 | 0.80 | 0.96 | 0.0041 | |
| Hospitalization for obesity (ref. Non) |  |  |  |  |  |  |  |  | 0.99 | 0.79 | 1.23 | 0.9024 | |
| Hospitalization for COPD (ref. Non) |  |  |  |  |  |  |  |  | 1.35 | 1.22 | 1.49 | <.0001 | |
| Hospitalization for alcoholism and related liver disorders (ref. Non) |  |  |  |  |  |  |  |  | 1.42 | 1.32 | 1.54 | <.0001 | |
| Hospitalization for depression (ref. Non) |  |  |  |  |  |  |  |  | 0.89 | 0.84 | 0.95 | 0.0006 | |
| Hospitalization for anxiety (ref. Non) |  |  |  |  |  |  |  |  | 0.94 | 0.87 | 1.01 | 0.0947 | |
| Hospitalization for hypertension (ref. Non) |  |  |  |  |  |  |  |  | 0.81 | 0.75 | 0.88 | <.0001 | |
| Hospitalization for diabetes (ref. Non) |  |  |  |  |  |  |  |  | 1.07 | 0.98 | 1.16 | 0.1278 | |
| Hospitalization for hyperlipidemia (ref. Non) |  |  |  |  |  |  |  |  | 0.85 | 0.63 | 1.15 | 0.2995 | |
| Hospitalization for tobacco use disorder (ref. Non) |  |  |  |  |  |  |  |  | 0.80 | 0.63 | 1.02 | 0.0697 | |
| HR: Hazard ratio; CI: Confidence interval; CHD: Coronary heart disease; COPD: Chronic obstructive pulmonary disease. | | | | | | | | | | | | | |
| Model 1: Univariate model, adjusted for age; Model 2: Adjusted for individual characteristics; Model 3: Full model. | | | | | | | | | | | | | |

| **Supplementary Table S6.** Hazards ratios (HR) and 95% confidence intervals (CI) for fatal CHD in women; Results of multivariable competing risk survival analysis | | | | | | | | | | | |  |
| --- | --- | --- | --- | --- | --- | --- | --- | --- | --- | --- | --- | --- |
|  | Model 1 | | |  | Model 2 | | |  | Model 3 | | |  |
|  | HR | 95% CI | |  | HR | 95% CI | |  | HR | 95% CI | | P-value |
| **Neighborhood deprivation** (ref. Low) |  |  |  |  |  |  |  |  |  |  |  |  |
| Moderate | 1.12 | 1.04 | 1.20 |  | 1.02 | 0.95 | 1.10 |  | 1.02 | 0.95 | 1.10 | 0.5305 |
| High | 1.52 | 1.40 | 1.65 |  | 1.31 | 1.21 | 1.43 |  | 1.30 | 1.19 | 1.41 | <.0001 |
| Age | 1.09 | 1.09 | 1.09 |  | 1.09 | 1.08 | 1.09 |  | 1.09 | 1.09 | 1.09 | <.0001 |
| Family income (ref. Highest quartiles) |  |  |  |  |  |  |  |  |  |  |  |  |
| Low |  |  |  |  | 1.57 | 1.43 | 1.73 |  | 1.53 | 1.39 | 1.69 | <.0001 |
| Middle-low |  |  |  |  | 1.40 | 1.27 | 1.54 |  | 1.35 | 1.22 | 1.49 | <.0001 |
| Middle-high |  |  |  |  | 1.22 | 1.10 | 1.35 |  | 1.20 | 1.08 | 1.33 | 0.0005 |
| Education attainment (ref. ≥ 12 years) |  |  |  |  |  |  |  |  |  |  |  |  |
| ≤ 9 years |  |  |  |  | 1.40 | 1.31 | 1.50 |  | 1.39 | 1.29 | 1.49 | <.0001 |
| 10–11 years |  |  |  |  | 1.11 | 1.03 | 1.19 |  | 1.11 | 1.03 | 1.19 | 0.0058 |
| Country of origin (ref. Sweden) |  |  |  |  | 0.91 | 0.84 | 1.00 |  | 0.91 | 0.84 | 0.99 | 0.0325 |
| Marital status (ref. Married/cohabiting) |  |  |  |  | 1.30 | 1.22 | 1.37 |  | 1.25 | 1.18 | 1.32 | <.0001 |
| Region of residence (ref. Large cities) |  |  |  |  |  |  |  |  |  |  |  |  |
| Middle-sized towns |  |  |  |  | 0.87 | 0.82 | 0.92 |  | 0.89 | 0.83 | 0.94 | <.0001 |
| Small towns/rural areas |  |  |  |  | 0.91 | 0.85 | 0.99 |  | 0.92 | 0.85 | 0.99 | 0.0349 |
| Hospitalization for obesity (ref. Non) |  |  |  |  |  |  |  |  | 1.02 | 0.88 | 1.18 | 0.7646 |
| Hospitalization for COPD (ref. Non) |  |  |  |  |  |  |  |  | 1.43 | 1.33 | 1.55 | <.0001 |
| Hospitalization for alcoholism and related liver disorders (ref. Non) |  |  |  |  |  |  |  |  | 1.70 | 1.55 | 1.87 | <.0001 |
| Hospitalization for depression (ref. Non) |  |  |  |  |  |  |  |  | 0.84 | 0.80 | 0.89 | <.0001 |
| Hospitalization for anxiety (ref. Non) |  |  |  |  |  |  |  |  | 0.89 | 0.83 | 0.95 | 0.0003 |
| Hospitalization for hypertension (ref. Non) |  |  |  |  |  |  |  |  | 0.80 | 0.74 | 0.85 | <.0001 |
| Hospitalization for diabetes (ref. Non) |  |  |  |  |  |  |  |  | 1.16 | 1.08 | 1.25 | <.0001 |
| Hospitalization for hyperlipidemia (ref. Non) |  |  |  |  |  |  |  |  | 0.88 | 0.69 | 1.14 | 0.3380 |
| Hospitalization for tobacco use disorder (ref. Non) |  |  |  |  |  |  |  |  | 0.85 | 0.69 | 1.04 | 0.1145 |
| HR: Hazard ratio; CI: Confidence interval; CHD: Coronary heart disease; COPD: Chronic obstructive pulmonary disease. | | | | | | | | | | | | |
| Model 1: Univariate model, adjusted for age; Model 2: Adjusted for individual characteristics; Model 3: Full model. | | | | | | | | | | | | |

| **Supplementary Table S7.** Hazard ratios (HR) and 95% confidence intervals (CI) for incident CHD; Results of Cox regression models | | | | | | | | |
| --- | --- | --- | --- | --- | --- | --- | --- | --- |
|  | Univariate model | | |  | Full model | | |  |
|  | HR | 95% CI | |  | HR | 95% CI | | P-value |
| **Neighborhood deprivation** (ref. Low) |  |  |  |  |  |  |  |  |
| Moderate | 1.16 | 1.07 | 1.26 |  | 1.11 | 1.02 | 1.21 | 0.0156 |
| High | 1.23 | 1.11 | 1.36 |  | 1.28 | 1.15 | 1.42 | <.0001 |
| Age | 1.06 | 1.06 | 1.07 |  | 1.06 | 1.06 | 1.07 | <.0001 |
| Gender to men (ref. Women) | 1.48 | 1.39 | 1.58 |  | 1.64 | 1.54 | 1.75 | <.0001 |
| Family income (ref. Highest quartiles) |  |  |  |  |  |  |  |  |
| Low | 0.85 | 0.78 | 0.94 |  | 0.89 | 0.80 | 0.98 | 0.0133 |
| Middle-low | 0.84 | 0.77 | 0.92 |  | 0.81 | 0.74 | 0.89 | <.0001 |
| Middle-high | 1.01 | 0.92 | 1.10 |  | 0.97 | 0.88 | 1.06 | 0.4770 |
| Education attainment (ref. ≥ 12 years) |  |  |  |  |  |  |  |  |
| ≤ 9 years | 1.95 | 1.81 | 2.10 |  | 1.20 | 1.10 | 1.30 | <.0001 |
| 10–11 years | 1.31 | 1.21 | 1.41 |  | 1.18 | 1.09 | 1.28 | <.0001 |
| Country of origin (ref. Sweden) | 0.97 | 0.89 | 1.07 |  | 1.18 | 1.07 | 1.29 | 0.0008 |
| Marital status (ref. Married/cohabiting) | 0.80 | 0.75 | 0.85 |  | 1.00 | 0.93 | 1.06 | 0.8863 |
| Region of residence (ref. Large cities) |  |  |  |  |  |  |  |  |
| Middle-sized towns | 0.95 | 0.88 | 1.02 |  | 0.93 | 0.87 | 1.00 | 0.0556 |
| Small towns/rural areas | 1.61 | 1.49 | 1.74 |  | 1.67 | 1.54 | 1.81 | <.0001 |
| Hospitalization for obesity (ref. Non) | 1.04 | 0.90 | 1.19 |  | 1.31 | 1.14 | 1.51 | 0.0001 |
| Hospitalization for COPD (ref. Non) | 1.77 | 1.62 | 1.94 |  | 1.44 | 1.31 | 1.58 | <.0001 |
| Hospitalization for alcoholism and related liver disorders (ref. Non) | 0.84 | 0.77 | 0.92 |  | 1.04 | 0.95 | 1.14 | 0.3797 |
| Hospitalization for depression (ref. Non) | 0.89 | 0.84 | 0.95 |  | 0.98 | 0.92 | 1.05 | 0.6249 |
| Hospitalization for anxiety (ref. Non) | 0.69 | 0.65 | 0.74 |  | 1.10 | 1.02 | 1.18 | 0.0136 |
| Hospitalization for hypertension (ref. Non) | 3.40 | 3.19 | 3.63 |  | 1.97 | 1.84 | 2.10 | <.0001 |
| Hospitalization for diabetes (ref. Non) | 2.48 | 2.30 | 2.67 |  | 1.60 | 1.48 | 1.72 | <.0001 |
| Hospitalization for hyperlipidemia (ref. Non) | 3.10 | 2.60 | 3.71 |  | 2.01 | 1.68 | 2.41 | <.0001 |
| Hospitalization for tobacco use disorder (ref. Non) | 2.00 | 1.71 | 2.34 |  | 2.18 | 1.86 | 2.56 | <.0001 |
| HR: Hazard ratio; CI: Confidence interval; CHD: Coronary heart disease; COPD: Chronic obstructive pulmonary disease. | | | | | | |  |  |

| **Supplementary Table S8.** Hazard ratios (HR) and 95% confidence intervals (CI) for fatal CHD; Results of multivariable competing risk survival analysis | | | | | | | | |
| --- | --- | --- | --- | --- | --- | --- | --- | --- |
|  | Univariate model | | |  | Full model | | |  |
|  | HR | 95% CI | |  | HR | 95% CI | | P-value |
| **Neighborhood deprivation** (ref. Low) |  |  |  |  |  |  |  |  |
| Moderate | 1.12 | 1.06 | 1.18 |  | 1.02 | 0.96 | 1.07 | 0.5465 |
| High | 1.37 | 1.29 | 1.45 |  | 1.31 | 1.23 | 1.40 | <.0001 |
| Age | 1.08 | 1.08 | 1.09 |  | 1.08 | 1.08 | 1.09 | <.0001 |
| Gender to men (ref. Women) | 1.17 | 1.12 | 1.21 |  | 1.46 | 1.40 | 1.52 | <.0001 |
| Family income (ref. Highest quartiles) |  |  |  |  |  |  |  |  |
| Low | 1.82 | 1.71 | 1.94 |  | 1.64 | 1.53 | 1.75 | <.0001 |
| Middle-low | 1.63 | 1.53 | 1.74 |  | 1.40 | 1.31 | 1.50 | <.0001 |
| Middle-high | 1.35 | 1.26 | 1.44 |  | 1.25 | 1.16 | 1.33 | <.0001 |
| Education attainment (ref. ≥ 12 years) |  |  |  |  |  |  |  |  |
| ≤ 9 years | 3.01 | 2.88 | 3.16 |  | 1.39 | 1.32 | 1.46 | <.0001 |
| 10–11 years | 1.31 | 1.25 | 1.38 |  | 1.12 | 1.06 | 1.18 | <.0001 |
| Country of origin (ref. Sweden) | 0.75 | 0.71 | 0.80 |  | 0.89 | 0.84 | 0.96 | 0.0008 |
| Marital status (ref. Married/cohabiting) | 1.07 | 1.03 | 1.11 |  | 1.27 | 1.22 | 1.33 | <.0001 |
| Region of residence (ref. Large cities) |  |  |  |  |  |  |  |  |
| Middle-sized towns | 0.89 | 0.85 | 0.93 |  | 0.87 | 0.84 | 0.91 | <.0001 |
| Small towns/rural areas | 0.87 | 0.82 | 0.92 |  | 0.90 | 0.85 | 0.95 | 0.0004 |
| Hospitalization for obesity (ref. Non) | 0.51 | 0.45 | 0.57 |  | 1.00 | 0.88 | 1.13 | 0.9841 |
| Hospitalization for COPD (ref. Non) | 1.64 | 1.55 | 1.74 |  | 1.40 | 1.32 | 1.48 | <.0001 |
| Hospitalization for alcoholism and related liver disorders (ref. Non) | 0.92 | 0.87 | 0.97 |  | 1.54 | 1.45 | 1.63 | <.0001 |
| Hospitalization for depression (ref. Non) | 0.71 | 0.68 | 0.73 |  | 0.86 | 0.83 | 0.90 | <.0001 |
| Hospitalization for anxiety (ref. Non) | 0.48 | 0.46 | 0.50 |  | 0.91 | 0.87 | 0.96 | 0.0002 |
| Hospitalization for hypertension (ref. Non) | 1.37 | 1.30 | 1.44 |  | 0.80 | 0.76 | 0.84 | <.0001 |
| Hospitalization for diabetes (ref. Non) | 1.47 | 1.40 | 1.56 |  | 1.12 | 1.06 | 1.18 | 0.0001 |
| Hospitalization for hyperlipidemia (ref. Non) | 0.92 | 0.76 | 1.11 |  | 0.87 | 0.72 | 1.06 | 0.1614 |
| Hospitalization for tobacco use disorder (ref. Non) | 0.74 | 0.64 | 0.87 |  | 0.82 | 0.70 | 0.96 | 0.0159 |
| HR: Hazard ratio; CI: Confidence interval; CHD: Coronary heart disease; COPD: Chronic obstructive pulmonary disease. | | | | | | |  |  |
